# Assessing the optimal virulence of malaria‐targeting mosquito pathogens: a mathematical study of engineered *Metarhizium anisopliae*

**DOI:** 10.1186/1475-2875-13-11

**Published:** 2014-01-08

**Authors:** Bernhard P Konrad, Michael Lindstrom, Anja Gumpinger, Jielin Zhu, Daniel Coombs

**Affiliations:** 1 Institute of Applied Mathematics and Department of Mathematics, University of British Columbia, 1984 Mathematics Road, Vancouver, BC, V6T 1Z2, Canada; 2 TU München, Fakultät für Mathematik, Boltzmannstraße 3, 85748 Garching (b. München), Germany

**Keywords:** Metarhizium anisopliae, Mathematical modelling, Fungal insecticide, Malaria control, Vector control

## Abstract

**Background:**

*Metarhizium anisopliae* is a naturally occurring fungal pathogen of mosquitoes. Recently, *Metarhizium* has been engineered to act against malaria by directly killing the disease agent within mosquito vectors and also effectively blocking onward transmission. It has been proposed that efforts should be made to minimize the virulence of the fungal pathogen, in order to slow the development of resistant mosquitoes following an actual deployment.

**Results:**

Two mathematical models were developed and analysed to examine the efficacy of the fungal pathogen. It was found that, in many plausible scenarios, the best effects are achieved with a reduced or minimal pathogen virulence, even if the likelihood of resistance to the fungus is negligible. The results for both models depend on the interplay between two main effects: the ability of the fungus to reduce the mosquito population, and the ability of fungus‐infected mosquitoes to compete for resources with non‐fungus‐infected mosquitoes.

**Conclusions:**

The results indicate that there is no obvious choice of virulence for engineered *Metarhizium* or similar pathogens, and that all available information regarding the population ecology of the combined mosquito‐fungus system should be carefully considered. The models provide a basic framework for examination of anti‐malarial mosquito pathogens that should be extended and improved as new laboratory and field data become available.

## Background

The major route of malaria transmission to humans is via blood feeding of female *Anopheles* mosquitoes (principally *Anopheles gambiae* and *Anopheles funestus*). Therefore, major efforts have been made to control mosquito populations in areas where malaria is prevalent. When first introduced, chemical insecticides were very efficient at reducing malaria prevalence in humans, although not without environmental damage. However, resistance has been observed to develop rapidly to broadly‐used insecticides and there is a lack of new chemical agents [1,2]. For this reason, fungal entomopathogens have been under extensive investigation as alternatives for mosquito control [3,4]. Diverse fungal pathogens of mosquitoes exist in nature and additionally can be genetically modified to generate desirable properties.

The focus of this paper is the application of engineered fungal pathogens of mosquitoes that can neutralize or kill malarial sporozoites in the mosquito vector itself, preventing onward transmission to humans. This is motivated by the recent development of an engineered (*Metarhizium anisopliae*) fungus strain [5]. *Metarhizium* is a natural parasite of mosquitoes that infects through direct contact with the insect cuticle, and therefore is appropriate for control strategies based on local spraying indoors or baited traps. Recombinant strains of *Metarhizium* have been designed to (i) block attachment of malarial sporozoites to salivary glands of the mosquito; and (ii) neutralize or kill *Plasmodium falciparum* directly within the mosquito hemolymph. Under laboratory conditions, these engineered pathogens were found to substantially reduce sporozoite counts in the salivary glands of mosquitoes, compared to both fungus‐uninfected mosquitoes and wild‐type *Metarhizium*[5]. This raises the possibility of producing a biological agent which targets the malaria parasite within the mosquito, and is thus able to disrupt the transmission cycle and reduce the prevalence of malaria in humans.

Interestingly, because fungal pathogens such as *Metarhizium* don’t kill infected mosquitoes until their later life‐stages, after the majority of mosquito reproduction has occurred, it is believed that the selection pressure for mosquito resistance is quite low and therefore resistance should develop slowly even under widespread deployment [6]. Mosquito resistance to fungal biopesticides of this type has not been reported to date. Nonetheless, the concern of emerging resistance leads Fang *et al.*[5] to argue against engineering *Metarhizium* to kill mosquitoes faster. This argument stands in contrast to a strategy where fungal species are applied in conjunction with chemical pesticides to reduce the overall numbers of mosquitoes (discussed in [3]).

However, even in the absence of developing resistance, an interesting question remains about how to optimize *Metarhizium* or similar agents in terms of virulence against mosquitoes: should one expect a high virulence agent to outperform an alternative low‐virulence strain? Indeed, if a high‐virulence mosquito pathogen strain has worse performance than a low‐virulence strain even in the absence of resistance, the threat of resistance additionally counts against it. (In this context, performance is measured in terms of reducing human malaria prevalence.) This paper presents simple mathematical models to investigate this question. It will be shown that the optimal choice of virulence level is not obvious, and depends quite sensitively on the details of the complex ecological system. In certain circumstances, it is expected to be preferable to apply a low‐virulence agent which penetrates the mosquito population, and is thus able to reduce the prevalence of malaria parasites in mosquitoes and humans, even though the overall population level of mosquitoes is not strongly impacted. Under other circumstances, a high‐virulence fungus that more effectively reduces the total mosquito population is expected to be preferable.

Mathematical modelling has been used in the study of malaria and as a tool for evaluating possible control strategies for over a century. The first mathematical models for malaria transmission were pioneered by Ross in 1911 [7] and developed further by Macdonald [8] and Anderson and May [9]. More recent mathematical models distinguish between exposed and infectious humans and mosquitoes reflect the fact that humans can become (temporarily) immune, treated or vaccinated, allow spatial heterogeneity, or include time‐dependent parameters to account for environmental factors such as rainfall and humidity (see for example [10]‐[13]). The current paper presents results from simple models for this new agent and potential improvements for future work are described.

## Methods

Two prototype mathematical models are proposed that take into account the most fundamental properties of the malaria parasite, transmission between human host and mosquito vector, and the fungal mosquito pathogen. To derive the models in a simple form, it is necessary to make a number of simplifying assumptions. These assumptions could be relaxed in future versions of the model, at the expense of analytical and intuitive understanding of the model results. Some possibilities for this work are described in the conclusions.

### Simplified model set‐up and assumptions

1. The total human population, *H*, is taken to be constant. This is roughly equivalent to supposing that the population dynamics of mosquitoes, malaria parasites and fungal pathogens equilibrate rapidly compared to the human demographic timescale. The (time‐dependent) fraction of humans that are able to infect mosquitoes with the malaria parasite is denoted by *h*(*t*) and these patients leave the infected group at rate *ρ* (for instance due to treatment, recovery or death). The human population is taken to be homogeneous in all other ways and the incubation period in the human host is assumed to be negligible compared to the duration of infectiousness.

2. The mosquito population is represented in three parts: uninfected (susceptible), infected with the malaria parasite, and fungus‐infected. These three populations are denoted by *S*(*t*), *I*(*t*), and *F*(*t*), respectively. It is necessary to make some simplifying assumptions concerning the population dynamics of mosquitoes, with and without the malaria parasite or the fungal pathogen. A commonly simplified model for biological populations is that of logistic growth [14]. This model has been applied in several previous works on malaria [12,15]‐[17]. Here, it is assumed that, in the absence of the malaria parasite and the fungus, the mosquito population experiences logistic growth with innate growth rate $\tilde{\kappa }$ and carrying capacity $\tilde{P}$. All mosquitoes also suffer a natural background death with rate denoted by *μ*, which is assumed to be independent of infection with the malaria parasite [18]. An alternative model of mosquito population dynamics, that includes larval and adult stages, is described below.

3. It is assumed that every mosquito has a human biting rate of *β*, irrespective of its infection status, and that if the human host is infectious, the bite always transmits the malaria parasite to the mosquito. On the other hand if an infected mosquito bites an uninfected human, the human will develop transmissible malaria with probability *γ*.

4. The fungal pathogen is constantly applied to the environment, and causes mosquitoes to transition from the *S* and *I* classes to the *F* class continuously, at constant rate *α*.

5. The fungal virulence (additional death of mosquitos due to fungal infection) is denoted by *σ*. To explicitly compare the natural mosquito death rate *μ* with the fungal virulence *σ*, one may rewrite the classical logistic growth equation $\text{dS}/\text{dt}=\tilde{\kappa }S(1-S/\tilde{P})$ in the form presented below, where $\kappa =\tilde{\kappa }+\mu$ and $P=\tilde{P}\kappa /(\kappa -\mu )$.

6. The fungus is assumed to be perfectly, permanently and immediately effective at blocking malaria parasite transmission to and from fungus‐infected mosquitoes.

Under these assumptions, the following system of ordinary differential equations can be put forward (*t* denotes time):

(1) $$\;\begin{array}{l} \frac{\text{dh}}{\text{dt}}=\beta \gamma \frac{I}{H}(1-h)-\mathrm{\rho h},\\ \frac{\text{dS}}{\text{dt}}=\kappa \left(S+I+F\right)\left(1-\frac{S+I+F}{P}\right)-\mathrm{\beta Sh}-(\mu +\alpha )S,\\ \frac{\text{dI}}{\text{dt}}=\mathrm{\beta Sh}-(\mu +\alpha )I,\\ \frac{\text{dF}}{\text{dt}}=\alpha (S+I)-(\mu +\sigma )\mathrm{F.} \end{array}$$

**Figure 1:**
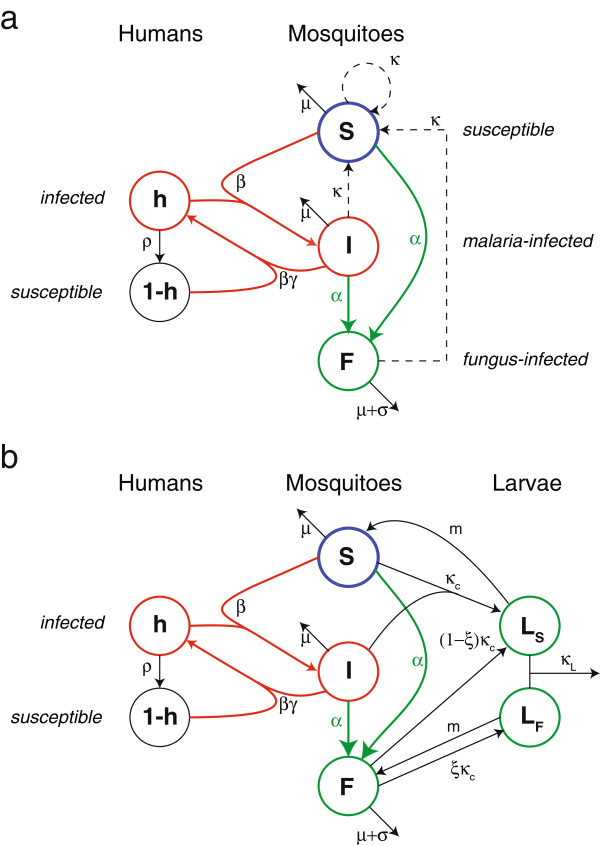
**Model schematics. a**. Simplified model. The fraction of infected humans in the population is given by *h*. Infection of susceptible mosquitoes *S* occurs at mass‐action rate *β* while removal/recovery occurs at a rate *ρ*. Infected mosquitoes *I* infect susceptible humans with probability *γ*, given an encounter. Malaria infection does not significantly alter the mosquito death rate *μ*, but fungus infection, occurring with rate *α* reflecting the intensity of fungal application, increases the death rate by *σ*. Since neither malaria nor the fungus is transmitted vertically in the simplified model all new‐born mosquitoes (indicated by *κ*) are susceptible. **b**. Life‐stage structured model. In this model mosquitoes produce larvae at rate *κ*_*c*_. Larvae may be fungal carriers (*L*_*F*_) or fungus‐free (*L*_*S*_) and undergo density‐dependent competition (see text) with intensity parameter *κ*_*L*_. Larvae mature to produce adult mosquitoes at rate *m*. The parameter *ξ* determines the degree of vertical transmissibility of the fungal pathogen.

A model schematic is given in Figure 1a.

### Life‐stage‐structured mosquito model and assumptions

Recent field studies strongly indicate that mosquito populations are controlled via density‐dependent regulation at the larval stage of development [19,20]. The simplified model above was derived under the assumption that these effects can be captured via a logistic model for the adult mosquito population, since the larval population is not modelled explicitly. This simplification allows for fairly clean analytical results, but the biological system may have been oversimplified. Additionally, vertical transmission is a feature of certain fungal symbionts of insects [21]. Therefore, a possible improvement of the engineered fungus would be a vertically transmissible variant, that could be passed from mosquitoes to their offspring. To accommodate the additional realism of larval competition for resources, and to consider vertical transmission, an alternative model is now put forward. This model is built under the following set of additional assumptions: 

1. Adult mosquitoes produce new larvae at a constant rate *κ*_*c*_, irrespective of infections with fungus or malaria parasites. The offspring of fungal‐infected mosquitoes are themselves infected with probability *ξ*, representing the probability of vertical transmission of the fungal pathogen. The populations of fungus‐uninfected and fungus‐infected larvae are denoted by *L*_*S*_(*t*) and *L*_*F*_(*t*) respectively. In the absence of information about larval infectibility, it is assumed that larvae are not directly infected by the fungus (although they may be infected via vertical transmission), and that the fungus has no detrimental effects in the larval stage.

2. Larvae compete for resources in a density‐dependent manner, with the intensity of competition determined by the parameter *κ*_*L*_.

3. Larvae mature into adult mosquitoes at rate *m*. In contrast to the simplified model, adult mosquitoes do not directly compete for resources.

Under these additional assumptions, a new life‐stage‐structured model can be put forward:

(2) $$\begin{array}{ll} \frac{\text{dh}}{\text{dt}} & =\mathrm{\beta \gamma I}\frac{\left(1-h\right)}{H}-\mathrm{\rho h}\\ \frac{{\text{dL}}_{S}}{\text{dt}} & =\kappa_{\text{c}}(S+I+(1-\xi )F)-\kappa_{L}\left(L_{S}+L_{F}\right)L_{S}-mL_{S}\\ \frac{{\text{dL}}_{F}}{\text{dt}} & =\kappa_{\text{c}}\mathrm{\xi F}-\kappa_{L}\left(L_{S}+L_{F}\right)L_{F}-mL_{F}\\ \frac{\text{dS}}{\text{dt}} & =mL_{S}-\mathrm{\beta Sh}-(\mu +\alpha )S\\ \frac{\text{dI}}{\text{dt}} & =\mathrm{\beta Sh}-(\mu +\alpha )I\\ \frac{\text{dF}}{\text{dt}} & =mL_{F}+\alpha (S+I)-(\mu +\sigma )F \end{array}$$

A model schematic is given in Figure 1b.

## Results and discussion

The goal is now to study the level of malaria in humans (*h*) as a function of the fungal parameters: the spraying rate *α*, fungal virulence *σ* and fungal vertical transmissibility *ξ*. For the simplified model, analytical results will be presented and applied to find the optimal values of *α* and *σ* in terms of reducing malaria in humans. For the life‐stage‐structured model, the situation is more complex and results will be shown only for particular numerical values of the parameters. In both models, it is found that application of a highly virulent fungus as biopesticide may not be the best strategy.

### The simplified model with no fungus

If there is no fungus present in the system, the simplified model simplifies to the following:

(3) $$\begin{array}{ll} \frac{\text{dh}}{\text{dt}} & =\beta \gamma \frac{I}{H}(1-h)-\mathrm{\rho h},\\ \frac{\text{dS}}{\text{dt}} & =\kappa \left(S+I\right)\left(1-\frac{S+I}{P}\right)-\mathrm{\beta Sh}-\mathrm{\mu S},\\ \frac{\text{dI}}{\text{dt}} & =\mathrm{\beta Sh}-\mathrm{\mu I.} \end{array}$$

This system has three relevant equilibria: (1) the trivial equilibrium (*h*,*S*,*I*)=(0,0,0) (no mosquitoes, no malaria); (2) the malaria‐free equilibrium $\left(h,S,I)=(0,(1-\frac{\mu }{\kappa })P,0\right)$ (mosquitoes but no malaria) and (3) the endemic equilibrium

(4) $$\;\begin{array}{l} (h,S,I)\;=\;\left(\;\frac{\beta^{2}\mathrm{\gamma P}(\kappa \;-\;\mu )\;-\;\mathrm{H\kappa \mu \rho}}{\beta (\mathrm{H\kappa \rho}\;+\;\mathrm{\beta \gamma P}(\kappa \;-\;\mu ))},\;\frac{\mu (\mathrm{H\kappa \rho}\;+\;\mathrm{\beta \gamma P}(\kappa \;-\;\mu ))}{\beta \gamma \kappa (\beta \;+\;\mu )},\right)\\ \;\left(\frac{\beta^{2}\mathrm{\gamma P}(\kappa -\mu )-\mathrm{H\kappa \mu \rho}}{\beta \gamma \kappa (\beta +\mu )}\right). \end{array}$$

From the malaria‐free equilibrium it is observed that *κ* > *μ* is the basic requirement for there to be mosquitoes in the system. This corresponds to the basic mosquito growth rate *κ* being large enough to outweigh mosquito death (and with no mosquitoes there can be no malaria).

The key quantity determining whether malaria will exist in the system is the basic reproduction number for the no‐fungus system, $R_{0}^{\text{(NF)}}=\beta \sqrt{\gamma S_{m}/(\mathrm{\rho \mu H})}$, where $S_{m}=P(1-\frac{\mu }{\kappa })$ is the mosquito population at the malaria‐free steady state. This quantity can be calculated using the next‐generation method [22]. The square root occurs in this formula because there are two steps per rounds of transmission, mosquito to human to mosquito, and the next‐generation *R*_0_ is, by definition, a per‐step quantity. The parameter *β* is not included in the square root because it determines the transmissibility human‐vector and vector‐human. It can also be shown that if $R_{0}^{\text{(NF)}}<1$ then the malaria‐free equilibrium is locally stable and the endemic equilibrium does not exist, while if $R_{0}^{\text{(NF)}}>1$ then the endemic equilibrium exists and is locally stable.

### Equilibrium analysis of the simplified model

The simplified model including the fungus (1) supports three distinctive equilibria, which can be defined as the *trivial*, *malaria‐free* and *endemic* equilibria. The following results on the local stability of these equilibria can then be proven (see Additional file 1 for the proofs, which are lengthy but straightforward): 

1. The trivial equilibrium (*h*,*S*,*I*,*F*)=(0,0,0,0) is locally asymptotically stable if and only if $\kappa <\frac{\left(\mu +\alpha )(\mu +\sigma \right)}{\mu +\alpha +\sigma }$. This condition is analogous to the condition *κ*>*μ* for the model with no fungus, described above.

2. The malaria‐free equilibrium

$$\;\begin{array}{l} (h_{m},S_{m},I_{m},F_{m})\;=\;\left(0,\frac{P(\mu \;+\;\sigma )}{\mu \;+\;\alpha \;+\;\sigma }\left(1\;-\;\frac{\left(\mu \;+\;\alpha )(\mu \;+\;\sigma \right)}{\kappa (\mu \;+\;\alpha \;+\;\sigma )}\right),\right)\\ \;\left(0,\frac{\mathrm{P\alpha}}{\mu \;+\;\alpha \;+\;\sigma }\left(1\;-\;\frac{\left(\;\mu \;+\;\alpha \;)(\;\mu \;+\;\sigma \;\right)}{\kappa (\;\mu \;+\;\alpha \;+\;\sigma \;)}\right)\;\right) \end{array}$$

exists in the positive plane and is locally asymptotically stable if and only if both of the following conditions are fulfilled:

$$\;\begin{array}{l} \kappa >\frac{\left(\mu +\alpha )(\mu +\sigma \right)}{\mu +\alpha +\sigma }\;\text{and}\;R_{0}=\beta \sqrt{\frac{\gamma S_{m}}{\rho (\mu +\alpha )H}}<1. \end{array}$$

The first condition corresponds to the survival of the mosquito population (exactly as for the trivial equilibrium). The second condition states that the basic reproductive number for malaria, *R*_0_, must be subcritical.

3. The endemic equilibrium

$$\;\begin{array}{l} (h_{e},S_{e},I_{e},F_{e})\;=\;\left(\;\frac{R_{0}^{2}\;-\;1}{R_{0}^{2}\;+\;\beta /(\mu \;+\;\alpha )},\;\left(1\;-\;\frac{\beta }{\mu \;+\;\alpha \;+\;\beta }\frac{R_{0}^{2}\;-\;1}{R_{0}^{2}}\right)\right)\\ \;\left(\;\times S_{m},\frac{\beta }{\mu +\alpha +\beta }\frac{R_{0}^{2}-1}{R_{0}^{2}}S_{m},F_{m}\right) \end{array}$$

exists in the positive plane and is locally asymptotically stable if and only if both of the following conditions are fulfilled:

$$\;\begin{array}{l} \kappa >\frac{\left(\mu +\alpha )(\mu +\sigma \right)}{\mu +\alpha +\sigma }\;\text{and}\;R_{0}=\beta \sqrt{\frac{\gamma S_{m}}{\rho (\mu +\alpha )H}}>1. \end{array}$$

By comparing *R*_0_ for the simplified model to the equivalent no‐fungus quantity ($R_{0}^{\text{(NF)}}$), it is possible to see the role of the spraying rate *α* in reducing the reproductive number of the malaria parasite. The virulence of the fungus (parameter *σ*) does not affect *R*_0_, because it is assumed that mosquitoes that are infected with the fungus are not able to transmit the malaria parasite.

### Optimizing the virulence and application of the fungal pathogen in the simplified model

**Figure 2:**
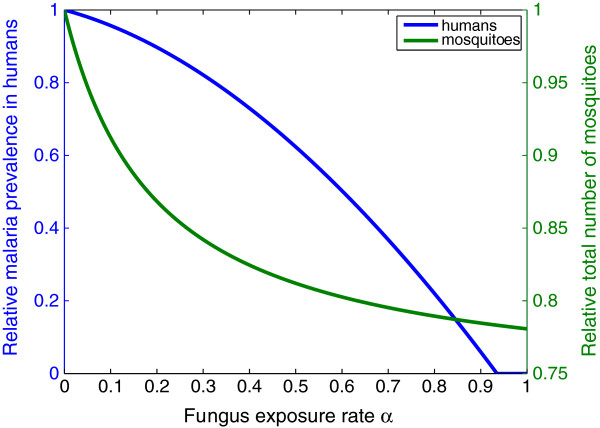
**Prevalence of malaria in humans and total number of mosquitoes for varying fungus deployment rates.** The steady state human malaria prevalence and the total mosquito population, both relative to baseline, are plotted against the fungus exposure intensity *α*, with fixed fungal pathogen virulence *σ*=0.1/day. As expected, quantities decrease when *α* increases. Note that, once *α* is large enough so that *R*_0_<1 and malaria is eradicated, *h*_*e*_ remains constant at 0. Full details of all other chosen parameter values are given in Additional file 2.

The two parameters of the model that are in principle under control in a real application are *α* (the application/spraying rate) and *σ* (the parameter controlling fungal virulence). Our goal is to choose *α* and *σ* such that the endemic malaria prevalence in humans

$$\begin{array}{l} h_{e}=h_{e}(\alpha ,\sigma )=\frac{R_{0}^{2}(\alpha ,\sigma )-1}{R_{0}^{2}(\alpha ,\sigma )+\beta /(\mu +\alpha )}, \end{array}$$

is minimized. The first and most intuitive result is that *h*_*e*_ is a monotonic decreasing function of *α*, which means that the more the mosquitoes are exposed to the fungus, the lower the endemic malaria prevalence is in humans. Increasing the fungus application rate is therefore always beneficial. This result is numerically illustrated in Figure 2. It can further be shown (see Additional file 1) that the total number of mosquitoes (*S*+*I*+*F*) also decreases when *α* increases, unless the fungal virulence *σ* is zero, in which case the total number of mosquitoes is independent of *α*.

There are two possible effects of continual fungus application that have to be considered in answering the question of how to find the optimal fungal virulence *σ*: 

1. *The biopesticide effect:* Increasing the fungal virulence will lead to a decrease in the total number of mosquitoes (*S*+*I*+*F*). This can be confirmed mathematically for the presented models (see Additional file 1).

2. *The competition effect:* Fungus‐infected mosquitoes *F* do not contribute directly to the transmission of the malaria parasite to humans. However, they do compete for resources with all the non‐fungus‐infected mosquitoes. Therefore, if the fungal virulence is low, then the population will have a higher fraction of non‐malaria‐carrying mosquitoes and the force of infection of the malaria parasite on humans could plausibly be reduced.

Hence there is a tradeoff between the two mechanisms above: the total number of mosquitoes could be minimized by a high‐virulence fungus (biopesticide effect), but what is critical for the prevalence in humans is the total number of mosquitoes infected with the malaria pathogen ‐ and this might be minimized (via the competition effect) by a low‐virulence fungus.

In order to determine the balance of these two effects, one must carefully analyse the mathematical model. It is then possible to establish a critical threshold for the relation between the mosquito innate growth rate *κ* and the natural mosquito death rate *μ* that makes one or the other argument stronger. Specifically, it can be shown that the malaria prevalence in the human population *h*_*e*_(*α*,*σ*) and the total number of infected mosquitoes *I*_*e*_(*α*,*σ*) are both *maximized* if

$$\begin{array}{l} \sigma =\sigma^{*}\equiv \frac{\left(\kappa -2\mu \right)}{2-\kappa /(\mu +\alpha )}. \end{array}$$

This result indicates how to best design the fungus: To avoid the worst case *σ*=*σ*^∗^, the virulence *σ* must be chosen far away from *σ*^∗^.

Note that while *σ*=*σ*^∗^ maximizes the malaria prevalence in humans as well as the total number of malaria‐infected mosquitoes, it does not maximize or minimize the total number of mosquitoes. This result reveals the complexity of the tradeoff between the biopesticide and the competition effect, and indicates the importance of a good understanding of the mosquito population as well as the fungus‐mosquito interaction, in designing the optimal pathogen for deployment.

### Optimal virulence depends on background mosquito growth rate

Continuing the analysis of the previous section, two interesting cases can be distinguished: (i) If *σ*^∗^ is negative, then *σ* is necessarily greater than *σ*^∗^ and hence virulence should always be *maximized* to reduce malaria prevalence in humans (indicating that the biopesticide effect is stronger than the competition effect); (ii) If *σ*^∗^ is positive, however, then a very large *σ* or a very low *σ* is desirable, but not an intermediate value. In the second case, this shows that the choice is either to use the fungal pathogen as a biopesticide (high virulence) or a distributed anti‐*Plasmodium* agent (low virulence) in the mosquito population.

The choice depends on the relation between the mosquito growth rate *κ*, the mosquito death rate *μ* and the fungal exposure rate *α*. If the growth rate *κ* is small compared to the mosquito death rate *μ* (specifically, if *κ*<2*μ*) then *σ*^∗^<0 for any value of *α*, and hence a larger fungal virulence *σ* is always desirable. This means that the fungus should be used as a biopesticide for slow‐growing mosquito populations. On the other hand, if the mosquito population is relatively fast‐growing (*κ*>2*μ*), then *σ*^∗^ is negative for 0<*α*<(*κ*−2*μ*)/2, while *σ*^∗^ is positive for *α*>(*κ*−2*μ*)/2. Parameter estimation (see Additional file 2) yields that *κ*≈5*μ*, implying that in most real mosquito populations, the latter scenario is much more realistic. Hence, if the fungus can be applied at a sufficient rate, then minimizing the fungal virulence *σ* has the most beneficial effect of malaria prevalence in humans.

**Figure 3:**
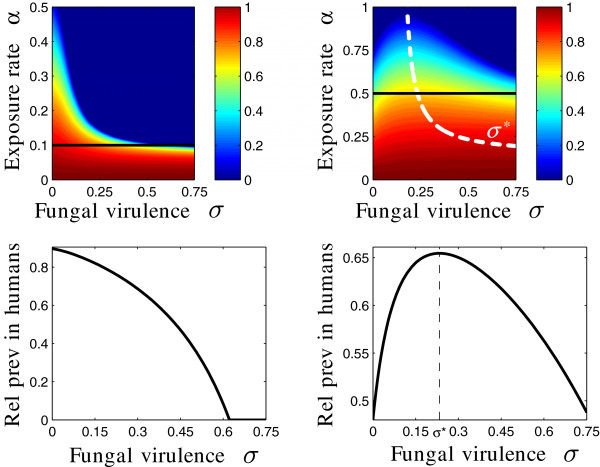
**Malaria prevalence in humans varying over fungal pathogen virulence and deployment rate.** The heat maps (top row) indicate the endemic malaria prevalence in humans *h*_*e*_, relative to baseline where no fungus is applied, for the given parameters, the graphs (bottom row) show a one‐dimensional projection of the heat maps, for a fixed fungal application (spraying) rate. Two distinct cases can be distinguished: (i) If *κ*−2*μ*<0 (left column) then human malaria prevalence is a decreasing function of fungal virulence *σ* and deployment rate *α*. (ii) If *κ*−2*μ*>0 (right column), the curve describing the worst‐case fungal virulence *σ*^∗^ is superimposed, indicating a non‐monotonic relationship between human malaria prevalence and *σ*. Top row: The dark blue region indicates *R*_0_<1 and hence *h*_*e*_=0. Bottom row: one‐dimensional slice through the heat map above for a fixed value of *α*=0.1/day (left) and *α*=0.5/day (right), as indicated by the black line in the heat map above. Here, *μ*=0.1/day, while *κ*=0.18/day (left) and *κ*=0.48/day (right). All other parameter values are as given in Additional file 2.

Figure 3 illustrates these principles for particular parameter choices. The human malaria prevalence *h*_*e*_(*α*,*σ*) is plotted, relative to the baseline prevalence when no fungus is applied, and hence indicating how malaria management can be optimized. The left column shows the slow‐growing result (*κ*<2*μ*) where the optimal strategy is to use the fungal pathogen as a biopesticide. The right column shows the more surprising result where, if the fungus deployment rate is sufficient, it is preferable to select a low‐virulence fungus strain. In all cases, verifying the first result of this section, it can be seen that a higher fungus‐exposure rate *α* is always desirable.

### Equilibrium analysis of the life‐stage‐structured model

The investigation of the simplified model has shown that depending on the ecology of the mosquito population, it can be expected that there are circumstances where the optimal virulence of the fungal pathogen can be zero. However, competition between mosquitoes in the simplified model takes place in the adult stage, but recent evidence indicates that competition occurs at the larval stage. To examine this effect it is necessary to use the more complex, life‐stage‐structured model (2), which includes the larval stage. In this section the effects of vertical transmission of fungus will also be examined.

### Life‐stage‐structured model without vertical transmission of fungus

By setting *ξ* = 0 in the second model, it is possible to investigate the model without vertical transmission. Similar to the simplified model, it can be shown that this model supports three easily explained equilibria, corresponding to (i) the trivial equilibrium with no mosquitoes and no malaria, (ii) a malaria‐free equilibrium with mosquitoes but no malaria, and (iii) an endemic equilibrium state. Provided that the malaria‐free equilibrium with mosquitoes exists, it can be shown that the endemic equilibrium state exists and is stable given the following reproductive number condition:

$$\begin{array}{l} R_{0}=\sqrt{\frac{\beta^{2}\gamma m^{2}((\kappa_{c}-\mu )(\mu +\alpha +\sigma )-\alpha \sigma )}{\kappa_{L}\rho {\left(\mu +\alpha \right)}^{3}(\mu +\sigma )H}}\\ R_{0}=\beta \sqrt{\frac{\gamma S_{0}}{\rho (\mu +\alpha )H}}>1, \end{array}$$

where

$$\begin{array}{l} S_{0}=\frac{m^{2}\left((\kappa_{c}-\mu )(\mu +\alpha +\sigma )-\alpha \sigma \right)}{\kappa_{L}{\left(\mu +\alpha \right)}^{2}(\mu +\sigma )} \end{array}$$

is the equilibrium number of mosquitoes in the population in the absence of the malaria parasite. Under this condition, the fraction of the human population infected with malaria, at the endemic equilibrium, is given by the same function of *R*_0_ as before (reflecting the very similar structures of the two models),

$$\begin{array}{l} h(\sigma )=\frac{R_{0}^{2}-1}{R_{0}^{2}+\beta /(\mu +\alpha )}. \end{array}$$

**Figure 4:**
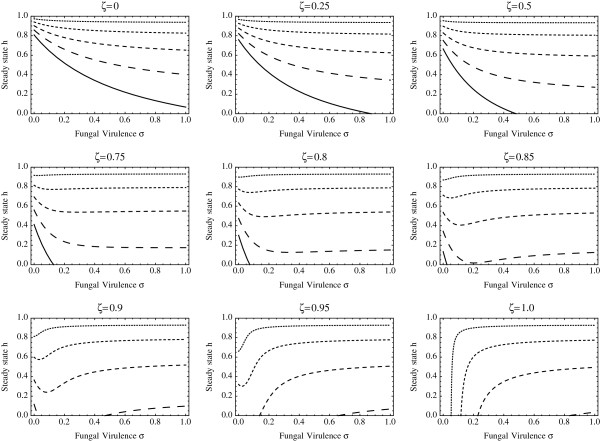
**Malaria prevalence in humans varying over vertical transmission, deployment rate and fungal virulence.** Each subfigure shows the endemic malaria prevalence in humans, relative to the no‐fungus baseline, plotted against the fungal virulence *σ*, for five different levels of the fungal application (spraying) rate *α*. The different values of *α* in each subfigure are {0.05,0.1,0.15,0.2,0.25} days ^−1^, corresponding to curves from top to bottom, and the length of the dashing increases with *α*. Each subfigure represents a different value of the vertical transmissibility *ξ* from *ξ*=0 (no vertical transmission) to *ξ*=1 (perfect vertical transmission). All other parameter values are as given in Additional file 2.

The derivative of this function with respect to *σ* is found to have no zeroes and is always negative for *R*_0_>1. This indicates that the human prevalence of malaria is a strictly decreasing function of *σ* and therefore *the best strategy for the life‐stage‐structured model with no vertical transmission of fungus is to increase the fungal virulence as much as possible*. This result (illustrated in Figure 4, top left panel where *ξ*=0) stands in contrast to the previous section where more nuanced conclusions were drawn, and is a consequence of the fact that the competition effect is occurring at the larval stage, while the biopesticide effect is occurring at the adult stage. Since it is adult mosquitoes, not larvae, that act as vectors for the malaria parasite, only the biopesticide effect can reduce malaria incidence in this version of the model.

### Life‐stage‐structured model with vertical transmission of fungus

Vertical transmission is a feature of fungal symbionts of insects [21] and it seems natural to consider this in the context of the model. The life‐stage‐structured model, where the parameter *ξ* is the vertical transmission fraction, reflects this possibility and allows the previous analysis to be repeated. In this case, the analytical expressions for the steady states of the full model can be obtained using a computer algebra system, such as Mathematica, but are too long to usefully give here. However, the basic analytical results remain: there are three potential equilibria of the model, but only one has mosquitoes and malaria. Further, although the model is resistant to analytical exploration, it is easy to choose parameters and work numerically.

Figure 4 shows the equilibrium fraction of infected humans as a function of the fungal virulence *σ* and application rate *α*, across a range of possible vertical transmission probabilities *ξ*. It is observed that if vertical transmission is unlikely then the benefit of the fungus is maximized by high virulence, but when vertical transmission becomes more likely, an intermediate or low virulence fungus would be favourable. In fact, in some cases a highly vertically transmissible fungus (*ξ*≥0.85 in Figure 4) is predicted to be able to eliminate malaria altogether, provided the virulence is low, but not too low. In all cases it should be noted that, as is to be expected, increasing the fungal application rate *α* is always beneficial.

These results indicate how the biopesticide effect of high virulence, acting at the adult stage, can be dominated by the competition effect at the larval stage, provided the adult and larval stages are coupled sufficiently strongly by vertical transmission of the fungus. In this case, highly virulent fungus are found to be less effective because they prevent the fungus‐infected mosquitoes from generating fungus‐carrying larvae. This reduces competitive inhibition of fungus‐uninfected larvae, thus reducing the overall effectiveness of the fungus in preventing malaria parasite infection of mosquitoes, and so ultimately increasing the prevalence of malaria in humans.

## Conclusions

In this paper, mathematical models have been employed to investigate the effectiveness of a fungal pathogen that blocks malaria transmission in mosquitoes to reduce malaria prevalence in humans. Unsurprisingly, all models indicate that malaria prevalence in humans could be reduced substantially by such a counter measure, and that the mosquito exposure rate to the fungus should be maximized to reduce malaria prevalence. However, the main interest of the results is to demonstrate that the optimal design of an agent that can simultaneously kill mosquitoes, *and malaria parasites within mosquitoes* depends quite sensitively on the details of a complex ecological system.

The first model, in which competition between mosquitoes occurred at the adult level, showed that in fast growing mosquito populations the fungal pathogen should be engineered to have low virulence. This result is independent of the possibility of mosquito resistance developing to the fungal pathogen and can be understood in very simple terms: fungus‐infected mosquitoes do not directly contribute to the malaria epidemic, but competitively hamper the introduction/survival of new susceptible mosquitoes. If mosquito resistance to fungal biopesticides arises in the field, this argument would additionally be strengthened.

In the second model, competition occurs at the larval stage of development. This model predicted that, in the absence of vertical transmission of the fungal pathogen, a highly virulent (biopesticidal) fungus would be desirable. However, the addition of reliable vertical transmission to this model significantly alters the predictions. If the fungal pathogen could be engineered in this way, then a high virulence would be highly detrimental to its efficacy as an anti‐malarial strategy.

The key observation that should be drawn is that finding the optimal properties of agents that can reduce the malaria parasite incidence in mosquitoes is not an easy task, since several direct and indirect effects need to be parameterized and balanced, leading to conclusions that depend on the details of the mosquito population, fungal pathogen, and environment.

In order to obtain a straightforward analytical treatment, several simplifying assumptions were made throughout. It was assumed that the fungus could completely block onward malaria transmission. Modifying this assumption in the simplified model leads to an increased favourability of virulence, but the authors believe that this issue will best be resolved in a future model where the time dependence of the fungus‐mosquito interaction is explicitly analysed. Also, by assuming a constant mass‐action biting coefficient, human reactions to avoid mosquito bites (bed nets or indoor residual spraying) were neglected. This was done to study the pure effect of the fungus interaction, but is likely not a realistic representation of a real‐life setting. Furthermore, the human population was modelled as a homogeneous population, neglecting for example co‐infections, age structure, and previous malaria history. All of the above have an important impact on the malaria epidemic: co‐infections increase the severity of each disease, children are much more vulnerable to malaria, and previous malaria infections can lead to temporal immunity. The incubation period of the malaria parasite and of the fungus in mosquitos were also neglected. This simplification particularly affects the mosquito population, where the incubation period is of about the same order as the life expectancy. Future work will include extending the current model to add more details of mosquito and malaria parasite life history in an age‐ and life‐stage‐dependent model of the mosquito.

Horizontal fungal transfer between mosquitoes was also not considered here. This effect has been observed for a number of fungal symbionts of insects and could play a role similar to vertical transmission in enhancing the effectiveness of the fungal pathogen. Horizontal transfer (possibly mediated through the environment) between mosquitoes would also be expected to increase the effectiveness of fungal spread, and reduce the necessary level of fungal application/spraying in the environment, which might otherwise have to be very intense. Further, in assuming a fairly simple logistic growth model, seasonal effects such as rainfall and humidity were neglected. These effects would lead to temporal variations in the mosquito population growth rate and carrying capacity, as well as unknown possible effects on the fungal pathogen. A final topic for possible future work is to consider spatial heterogeneities such as breeding sites and human habitat.

Despite these limitations, the models are useful in defining an argument for a minimal virulence of the antimalarial fungal pathogens. In future work, as field studies of *Metarhizium* or similar agents are completed, the parameterization of the model can be improved, and new models that allow insights into the potential of a large‐scale deployment of such controls for malaria and other mosquito‐borne diseases can be developed.

## Competing interests

The authors declare that they have no competing interests.

## Authors’ contributions

Established the study: BPK, ML, JZ, DC. Developed and analysed the models: BPK, ML, JZ, AG, DC. Wrote the paper: BPK, DC. All authors have read and approved the final version.

## Supplementary Material

Additional file 1 Contains full details of mathematical proofs for some statements made in the main text. Click here for file

Additional file 2 Includes details of parameter estimation used in generating the figures. Click here for file
