# Supplementary material for: Assessing the optimal virulence of malaria‐targeting mosquito pathogens: a mathematical study of engineered Metarhizium anisopliae
Source: Malar J. 2014 Jan 8;13:11. doi: 10.1186/1475-2875-13-11 (PMC3893513; doi:10.1186/1475-2875-13-11)
Supplement: Additional file 1 — Contains full details of mathematical proofs for some statements made in the main text. [file 1475-2875-13-11-S1.pdf]

Additional file 1 for: Assessing the optimal virulence of malaria-targeting mosquito pathogens: a mathematical study of engineered *Metarhizium anisopliae*

Bernhard Konrad[1], Michael Lindstrom[1], Anja Gumpinger[2], Jielin Zhu[1], Daniel Coombs[1,*]

1 Department of Mathematics and Institute of Applied Mathematics, University of British Columbia, Vancouver, BC, Canada

2 TU München, Fakultät für Mathematik, Boltzmannstraße 3, 85748 Garching (b. München), Germany

***E-mail: coombs@math.ubc.ca**

Proof of theorems

In this additional file we present the lengthy but straightforward proofs of some mathematical results used in the text.

Main Result 1 (Local Stability). *1. The trivial equilibrium $(h, S, I, F) = (0, 0, 0, 0)$ is locally asymptotically stable if and only if $\kappa < \frac{(\mu+\alpha)(\mu+\sigma)}{\mu+\alpha+\sigma}$.*

2. The malaria-free equilibrium $(h, S, I, F) = (0, S_m, 0, F_m)$ exists in the positive plane and is locally asymptotically stable if and only if both of the following conditions are fulfilled:

$$\kappa > \frac{(\mu+\alpha)(\mu+\sigma)}{\mu+\alpha+\sigma} \quad \textit{and} \quad R_0 = \beta\sqrt{\frac{\gamma S_m}{\rho(\mu+\alpha)H}} < 1.$$

Where $S_m = \frac{P(\mu+\sigma)}{\mu+\alpha+\sigma}(1 - \frac{(\mu+\alpha)(\mu+\sigma)}{\kappa(\mu+\alpha+\sigma)})$ and $F_m = \frac{\alpha}{\mu+\sigma}S_m$.

3. The endemic equilibrium $(h, S, I, F) = (h_e, S_e, I_e, F_e)$ exists in the positive plane and is locally asymptotically stable if and only if both of the following conditions are fulfilled:

$$\kappa > \frac{(\mu+\alpha)(\mu+\sigma)}{\mu+\alpha+\sigma} \quad \textit{and} \quad R_0 = \beta\sqrt{\frac{\gamma S_m}{\rho(\mu+\alpha)H}} > 1.$$

Where $h_e = \frac{R_0^2-1}{R_0^2+\beta/(\mu+\alpha)}$, $S_e = (1 - \frac{\beta}{\mu+\alpha+\beta}\frac{R_0^2-1}{R_0^2})S_m$, $I_e = \frac{\beta}{\mu+\alpha+\beta}\frac{R_0^2-1}{R_0^2}S_m$, $F_e = F_m$.

Proof. We first show how to get to the steady states and that the system indeed has exactly these three steady states. The trivial steady state $(h, S, I, F) = (0, 0, 0, 0)$ is straight forward.

In the malaria-free steady state neither infected humans ($h_m = 0$), nor infected mosquitoes ($I_m = 0$) are present, but susceptible mosquitoes S_m and mosquitoes with fungus F_m are around. The equations reduce to

$$\frac{dS}{dt} = \kappa(S+F)\left(1 - \frac{S+F}{P}\right) - (\mu+\alpha)S$$
$$\frac{dF}{dt} = \alpha S - (\mu+\sigma)F$$

Setting the rate of change $\frac{dF}{dt}$ to zero tells us $F_m = \frac{\alpha}{\mu+\sigma}S_m$. Plugging this into the former equation, after

we set $\frac{dS}{dt}$ to zero yields (note that $S_m + F_m = \frac{\mu+\alpha+\sigma}{\mu+\sigma} S_m$)

$$
\begin{aligned}
0 &= \kappa S_m \frac{\mu+\alpha+\sigma}{\mu+\sigma}\left(1 - \frac{\mu+\alpha+\sigma}{\mu+\sigma}\frac{S_m}{P}\right) - (\mu+\alpha) S_m \\
0 &= \frac{\kappa(\mu+\alpha+\sigma)}{\mu+\sigma} - \frac{\kappa(\mu+\alpha+\sigma)^2}{P(\mu+\sigma)^2} S_m - \frac{(\mu+\alpha)(\mu+\sigma)}{\mu+\sigma} \\
S_m &= \frac{P(\mu+\sigma)(\kappa(\mu+\alpha+\sigma) - (\mu+\alpha)(\mu+\sigma))}{\kappa(\mu+\alpha+\sigma)^2} \\
&= \frac{P(\mu+\sigma)}{\mu+\alpha+\sigma}\left(1 - \frac{(\mu+\alpha)(\mu+\sigma)}{\kappa(\mu+\alpha+\sigma)}\right),
\end{aligned}
$$

where we divided by S_m since we assumed $S_m \neq 0$. If $S_m = 0$ we reach the trivial steady state. Therefore

$$
F_m = \frac{\alpha}{\mu+\sigma} S_m = \frac{\alpha P}{\mu+\alpha+\sigma}\left(1 - \frac{(\mu+\alpha)(\mu+\sigma)}{\kappa(\mu+\alpha+\sigma)}\right).
$$

This brings us to the endemic steady state, where h_e, S_e, I_e and F_e are all distinct from zero. (Note that $S_e = 0$ or $F_e = 0$ would lead us to the trivial steady state, since populations can not be negative.)

First, note that we can divide by $1 - h_e$, since $h_e \neq 1$ due to the first equation in (1). Setting $\frac{dh}{dt} = 0$ yields $I_e = \frac{\rho H h_e}{\beta\gamma(1-h_e)}$. Setting $\frac{dI}{dt} = 0$ yields $I_e = \frac{\beta S_e h_e}{\mu+\alpha}$. Combining those those two equations yields $S_e = \frac{\rho H(\mu+\alpha)}{\beta^2\gamma(1-h_e)}$. Next, setting $\frac{dF}{dt} = 0$ we obtain

$$
F_e = \frac{\alpha}{\mu+\sigma}(S_e + I_e) = \frac{\alpha\rho H}{\beta^2\gamma(\mu+\sigma)(1-h_e)}(\beta h_e + \mu + \alpha).
$$

Therefore,

$$
\begin{aligned}
S_e + I_e + F_e &= \frac{\rho H}{\beta\gamma(1-h_e)}\left(\frac{\mu+\alpha}{\beta} + h_e + \frac{\alpha}{\beta(\mu+\sigma)}(\beta h_e + \mu + \alpha)\right) \\
&= \frac{\rho H}{\beta^2\gamma(1-h_e)}\left(\mu + \alpha + \beta h_e + \frac{\alpha}{\mu+\sigma}(\beta h_e + \mu + \alpha)\right) \\
&= \frac{\rho H(\mu+\alpha+\sigma)}{\beta^2\gamma(\mu+\sigma)(1-h_e)}(\beta h_e + \mu + \alpha)
\end{aligned}
$$

Plugging this into the equation $\frac{dS}{dt} = 0$ will lead to h_e, but not before a lengthy calculation:

$$
\begin{aligned}
0 = \kappa &\frac{\rho H(\mu+\alpha+\sigma)}{\beta^2\gamma(\mu+\sigma)(1-h_e)}(\beta h_e + \mu + \alpha)\left(1 - \frac{\rho H(\mu+\alpha+\sigma)}{P\beta^2\gamma(\mu+\sigma)(1-h_e)}(\beta h_e + \mu + \alpha)\right) \\
&- \beta h_e \frac{\rho H(\mu+\alpha)}{\beta^2\gamma(1-h_e)} - (\mu+\alpha)\frac{\rho H(\mu+\alpha)}{\beta^2\gamma(1-h_e)}
\end{aligned}
$$

multiplying by $\frac{\beta^2\gamma(1-h_e)}{\rho H}$ yields

$$
0 = \frac{\kappa(\mu+\alpha+\sigma)}{\mu+\sigma}(\beta h_e + \mu + \alpha)\left(1 - \frac{\rho H(\mu+\alpha+\sigma)}{P\beta^2\gamma(\mu+\sigma)(1-h_e)}(\beta h_e + \mu + \alpha)\right) - (\mu+\alpha)(\beta h_e + \mu + \alpha).
$$

Multiplying by $\frac{\mu+\sigma}{\kappa(\mu+\alpha+\sigma)(\beta h_e+\mu+\alpha)}$, which is positive since h_e is positive, and rearranging we obtain

$$
\frac{\rho H(\mu+\alpha+\sigma)(\beta h_e + \mu + \alpha)}{P\beta^2\gamma(\mu+\sigma)(1-h_e)} = 1 - \frac{(\mu+\alpha)(\mu+\sigma)}{\kappa(\mu+\alpha+\sigma)}
$$

To solve this for h_e we multiply by the denominator on the left hand side and expand the resulting terms:

$$
\begin{aligned}
\rho H(\mu+\alpha+\sigma)\beta h_e + \rho H(\mu+\alpha+\sigma)(\mu+\alpha) &= P\beta^2\gamma(\mu+\sigma) - P\beta^2\gamma(\mu+\sigma)h_e \\
&\quad - \frac{P\beta^2\gamma(\mu+\alpha)(\mu+\sigma)^2}{\kappa(\mu+\alpha+\sigma)} + \frac{P\beta^2\gamma(\mu+\alpha)(\mu+\sigma)^2}{\kappa(\mu+\alpha+\sigma)} h_e,
\end{aligned}
$$

which we rearrange to

$$h_e\left[\rho\beta H(\mu+\alpha+\sigma)+P\beta^2\gamma(\mu+\sigma)\left(1-\frac{(\mu+\alpha)(\mu+\sigma)}{\kappa(\mu+\alpha+\sigma)}\right)\right]$$
$$=P\beta^2\gamma(\mu+\sigma)\left(1-\frac{(\mu+\alpha)(\mu+\sigma)}{\kappa(\mu+\alpha+\sigma)}\right)-\rho H(\mu+\alpha+\sigma)(\mu+\alpha).$$

We now divide by $\rho H(\mu+\alpha)(\mu+\alpha+\sigma)$ and recall that $R_0^2=\frac{\beta^2\gamma S_m}{\rho(\mu+\alpha)H}=\frac{P\beta^2\gamma(\mu+\sigma)}{\rho H(\mu+\alpha)(\mu+\alpha+\sigma)}(1-\frac{(\mu+\alpha)(\mu+\sigma)}{\kappa(\mu+\alpha+\sigma)})$. This results in

$$h_e\left(R_0^2+\frac{\beta}{\mu+\alpha}\right)=R_0^2-1,\quad \text{or equivalently}\quad h_e=\frac{R_0^2-1}{R_0^2+\beta/(\mu+\alpha)}.$$

From here we can quickly find S_e, I_e and F_e:

$$S_e=\frac{\rho H(\mu+\alpha)}{\beta^2\gamma(1-h_e)}=\frac{S_m}{R_0^2(1-h_e)}=\frac{S_m}{R_0^2}\frac{R_0^2+\frac{\beta}{\mu+\alpha}}{1+\frac{\beta}{\mu+\alpha}}=S_m\left(1-\frac{\beta}{\beta+\mu+\alpha}\frac{R_0^2-1}{R_0^2}\right)$$
$$I_e=\frac{\rho H h_e}{\beta\gamma(1-h_e)}=\frac{\beta S_m}{R_0^2(\mu+\alpha)}\frac{h_e}{1-h_e}=\frac{\beta S_m}{R_0^2(\mu+\alpha)}\frac{R_0^2-1}{R_0^2+\frac{\beta}{\mu+\alpha}}\frac{R_0^2+\frac{\beta}{\mu+\alpha}}{1+\frac{\beta}{\mu+\alpha}}$$
$$=S_m\frac{\beta}{\beta+\mu+\alpha}\frac{R_0^2-1}{R_0^2}$$
$$F_e=\frac{\alpha}{\mu+\sigma}(S_e+I_e)=\frac{\alpha}{\mu+\sigma}S_m=F_m$$

To determine the local stability of the steady states we compute the characteristic polynomial of the linearized system around the equilibrium:

$$\det\begin{vmatrix}-\beta\gamma\frac{I}{H}-\rho-\lambda & 0 & \beta\gamma\frac{1}{H}(1-h) & 0\\ -\beta S & \kappa(1-\frac{2(S+I+F)}{P})-\beta h-(\mu+\alpha)-\lambda & \kappa(1-\frac{2(S+I+F)}{P}) & \kappa(1-\frac{2(S+I+F)}{P})\\ \beta S & \beta h & -(\mu+\alpha)-\lambda & 0\\ 0 & \alpha & \alpha & -(\mu+\sigma)-\lambda\end{vmatrix}$$

$$=\det\begin{vmatrix}-\beta\gamma\frac{I}{H}-\rho-\lambda & 0 & \beta\gamma\frac{1}{H}(1-h) & 0\\ 0 & \kappa(1-\frac{2(S+I+F)}{P})-(\mu+\alpha)-\lambda & \kappa(1-\frac{2(S+I+F)}{P})-(\mu+\alpha)-\lambda & \kappa(1-\frac{2(S+I+F)}{P})\\ \beta S & \beta h & -(\mu+\alpha)-\lambda & 0\\ 0 & \alpha & \alpha & -(\mu+\sigma)-\lambda\end{vmatrix}$$

$$=\det\begin{vmatrix}-\beta\gamma\frac{I}{H}-\rho-\lambda & 0 & \beta\gamma\frac{1}{H}(1-h) & 0\\ 0 & \kappa(1-\frac{2(S+I+F)}{P})-(\mu+\alpha)-\lambda & 0 & \kappa(1-\frac{2(S+I+F)}{P})\\ \beta S & \beta h & -(\mu+\alpha)-\beta h-\lambda & 0\\ 0 & \alpha & 0 & -(\mu+\sigma)-\lambda\end{vmatrix}$$

$$=\underbrace{\det\begin{vmatrix}-\beta\gamma\frac{I}{H}-\rho-\lambda & \beta\gamma\frac{1}{H}(1-h)\\ \beta S & -(\mu+\alpha)-\beta h-\lambda\end{vmatrix}}_{=:A}\underbrace{\det\begin{vmatrix}\kappa\left(1-\frac{2(S+I+F)}{P}\right)-(\mu+\alpha)-\lambda & \kappa\left(1-\frac{2(S+I+F)}{P}\right)\\ \alpha & -(\mu+\sigma)-\lambda\end{vmatrix}}_{=:B}$$

The fourth-order polynomial factors nicely into two second-order polynomials. This is to be expected: Polynomial A says if malaria can invade the population, polynomial B decides if mosquitoes can invade the environment.

We now split the analysis into the three equilibria:

1. **Trivial equilibrium** $(h, S, I, F) = (0, 0, 0, 0)$**.** Then A becomes

$$A = \det \begin{vmatrix} -\rho - \lambda & \beta\gamma\frac{1}{H} \\ 0 & -(\mu+\alpha) - \lambda \end{vmatrix} = (\rho + \lambda)(\mu + \alpha + \lambda),$$

which clearly has the two negative roots $\lambda = -\rho$ and $\lambda = -(\mu + \alpha)$. Hence, malaria can not invade a void population and the stability of the trivial steady state is solely determined by B.

B becomes

$$\begin{aligned} B &= \begin{vmatrix} \kappa - (\mu+\alpha) - \lambda & \kappa \\ \alpha & -(\mu+\sigma) - \lambda \end{vmatrix} \\ &= ((\kappa - (\mu+\alpha)) - \lambda)((-(\mu+\sigma)) - \lambda) - \alpha\kappa \\ &= \lambda^2 + [(\mu+\sigma) + (\mu+\alpha) - \kappa]\,\lambda + (\mu+\alpha)(\mu+\sigma) - \kappa(\alpha+\mu+\sigma) \end{aligned}$$

The constant term is positive if and only if

$$\kappa < \frac{(\mu+\alpha)(\mu+\sigma)}{\mu+\alpha+\sigma} = (\mu+\alpha)\frac{\mu+\sigma}{\mu+\alpha+\sigma} \tag{1}$$

If this condition is not fulfilled, then there is a positive root and hence the steady state is not stable. If this condition is fulfilled, then $\kappa < (\mu + \alpha) < (\mu + \alpha) + (\mu + \sigma)$ and hence the linear term is also positive. Thus only negative roots exist and the steady state is table. Hence the trivial steady state is stable if and only if the condition (1) on κ holds.

2. **Malaria-free equilibrium** $(h, S, I, F) = (0, S_m, 0, F_m)$**.**

   In the malaria-free equilibrium A becomes

$$\begin{aligned} A = \det \begin{vmatrix} -\rho - \lambda & \beta\gamma\frac{1}{H} \\ \beta S_m & -(\mu+\alpha) - \lambda \end{vmatrix} &= (-\rho - \lambda)(-(\mu+\alpha) - \lambda) - \beta^2\gamma\frac{S_m}{H} \\ &= \lambda^2 + (\rho + \mu + \alpha)\,\lambda + \rho(\mu+\alpha) - \beta^2\gamma\frac{S_m}{H}. \end{aligned}$$

   This polynomial has only negative roots if and only if the constant term is positive, i.e. if and only if

$$\rho(\mu+\alpha) - \beta^2\gamma\frac{S_m}{H} > 0,$$

   which is equivalent to $R_0 = \beta\sqrt{\frac{\gamma S_m}{\rho(\mu+\alpha)H}} < 1$.

   The polynomial B becomes

$$B = \det \begin{vmatrix} \kappa\left(1 - \frac{2(S_m+F_m)}{P}\right) - (\mu+\alpha) - \lambda & \kappa\left(1 - \frac{2(S_m+F_m)}{P}\right) \\ \alpha & -(\mu+\sigma) - \lambda \end{vmatrix}$$

   Before we go about calculating this, let us first simplify the first row a little bit:

$$\begin{aligned} S_m + F_m = S_m + \frac{\alpha}{\mu+\sigma}S_m &= \frac{\mu+\alpha+\sigma}{\mu+\sigma}S_m \\ &= P\frac{\kappa(\mu+\alpha+\sigma) - (\mu+\alpha)(\mu+\sigma)}{\kappa(\mu+\alpha+\sigma)} \\ &= P\left(1 - \frac{(\mu+\alpha)(\mu+\sigma)}{\kappa(\mu+\alpha+\sigma)}\right) \end{aligned}$$

   With that we obtain

$$\kappa\left(1 - \frac{2(S_m+F_m)}{P}\right) = \kappa\left(1 - 2\left(1 - \frac{(\mu+\alpha)(\mu+\sigma)}{\kappa(\mu+\alpha+\sigma)}\right)\right) = 2\frac{(\mu+\alpha)(\mu+\sigma)}{\mu+\alpha+\sigma} - \kappa.$$

Therefore

$$
\begin{aligned}
B &= \det \begin{vmatrix} \kappa\left(1-\frac{2(S_m+F_m)}{P}\right)-(\mu+\alpha)-\lambda & \kappa\left(1-\frac{2(S_m+F_m)}{P}\right) \\ \alpha & -(\mu+\sigma)-\lambda \end{vmatrix} \\
&= \det \begin{vmatrix} 2\frac{(\mu+\alpha)(\mu+\sigma)}{\mu+\alpha+\sigma}-\kappa-(\mu+\alpha)-\lambda & 2\frac{(\mu+\alpha)(\mu+\sigma)}{\mu+\alpha+\sigma}-\kappa \\ \alpha & -(\mu+\sigma)-\lambda \end{vmatrix} \\
&= \left(2\frac{(\mu+\alpha)(\mu+\sigma)}{\mu+\alpha+\sigma}-\kappa-(\mu+\alpha)-\lambda\right)(-(\mu+\sigma)-\lambda)-\alpha\left(2\frac{(\mu+\alpha)(\mu+\sigma)}{\mu+\alpha+\sigma}-\kappa\right) \\
&= \lambda^2+\lambda\left[\kappa+(\mu+\alpha)+(\mu+\sigma)-2\frac{(\mu+\alpha)(\mu+\sigma)}{\mu+\alpha+\sigma}\right] \\
&\quad +\kappa(\mu+\alpha+\sigma)+(\mu+\alpha)(\mu+\sigma)-2\frac{(\mu+\alpha)(\mu+\sigma)}{\mu+\alpha+\sigma}(\mu+\alpha+\sigma) \\
&= \lambda^2+\lambda\left[\kappa+(\mu+\alpha)\left(1-\frac{\mu+\sigma}{\mu+\alpha+\sigma}\right)+(\mu+\sigma)\left(1-\frac{\mu+\alpha}{\mu+\alpha+\sigma}\right)\right] \\
&\quad +\kappa(\mu+\alpha+\sigma)-(\mu+\alpha)(\mu+\sigma).
\end{aligned}
$$

It is now obvious that this polynomial has two roots with negative real part, if only if the constant term is positive, i.e. if

$$\kappa > \frac{(\mu+\alpha)(\mu+\sigma)}{\mu+\alpha+\sigma}.$$

3. **Endemic equilibrium** $(h,S,I,F)=(h_e,S_e,I_e,F_e)$**.**

In the endemic steady state the polynomial A becomes

$$
\begin{aligned}
A &= \begin{vmatrix} -\beta\gamma\frac{I_e}{H}-\rho-\lambda & \beta\gamma\frac{1}{H}(1-h_e) \\ \beta S_e & -(\mu+\alpha)-\beta h_e-\lambda \end{vmatrix} \\
&= \lambda^2+\lambda\left[\beta\gamma\frac{I_e}{H}+\rho+\mu+\alpha+\beta h_e\right] \\
&\quad +\beta\gamma\frac{I_e}{H}(\mu+\alpha)+\beta^2\gamma\frac{I_e}{H}h_e+\rho(\mu+\alpha)+\beta\rho h_e-\beta^2\gamma\frac{S_e}{H}+\beta^2\gamma\frac{S_e}{H}h_e \\
&= \lambda^2+\lambda\left[\beta\gamma\frac{I_e}{H}+\rho+\mu+\alpha+\beta h_e\right] \\
&\quad +\beta\gamma\frac{I_e}{H}(\mu+\alpha)+\rho(\mu+\alpha)+\beta^2\gamma\frac{I_e}{H}-\beta^2\gamma\frac{S_e}{H}(1-h_e),
\end{aligned}
$$

where we used that $\rho h_e=\beta\gamma\frac{I_e}{H}(1-h_e)$. In the constant term we now substitute S_e and I_e with the expressions we just found above, and also use the definition of R_0. Factoring the result yields

$$
\begin{aligned}
A &= \lambda^2+\lambda\left[\beta\gamma\frac{I_e}{H}+\rho+\mu+\alpha+\beta h_e\right] \\
&\quad +\frac{\beta^2\gamma S_m}{H}\left(\frac{\mu+\alpha}{\beta+\mu+\alpha}\frac{R_0^2-1}{R_0^2}+\frac{1}{R_0^2}+\frac{\beta}{\beta+\mu+\alpha}\frac{R_0^2-1}{R_0^2}-(1-h_e)\left(1-\frac{\beta}{\beta+\mu+\alpha}\frac{R_0^2-1}{R_0^2}\right)\right) \\
&= \lambda^2+\lambda\left[\beta\gamma\frac{I_e}{H}+\rho+\mu+\alpha+\beta h_e\right]+R_0^2\rho(\mu+\alpha)\left(h_e+(1-h_e)\frac{\beta}{\beta+\mu+\alpha}\frac{R_0^2-1}{R_0^2}\right),
\end{aligned}
$$

where we again used the definition of R_0. We now see that, if $R_0>1$, then all roots of A have negative real part.

For B, note that $S_e+I_e+F_e=S_m+F_m$, and hence B is the same as in the malaria-free steady state. □

Main Result 2 (Fungus exposure rate)**.** *The malaria prevalence in humans $h_e = h_e(\alpha, \sigma)$ decreases when the fungus-exposure rate α increases. The total number of mosquitoes $S_e + I_e + F_e = (S_e + I_e + F_e)(\alpha, \sigma)$ also decreases when α increases, unless the fungus-induced death rate σ is zero, in which case the total number of mosquitoes is unaffected by α.*

Proof. Before we prove the claim, we first show that $S_m = S_m(\alpha, \sigma)$ is a decreasing function of α is, i.e.

$$\frac{\partial S_m}{\partial \alpha}(\alpha, \sigma) < 0$$

for all $\alpha, \sigma \geq 0$. A straight forward calculation yields

$$\begin{aligned}
\frac{\partial S_m}{\partial \alpha}(\alpha, \sigma) &= \frac{\partial}{\partial \alpha}\left[\frac{P(\mu+\sigma)}{\mu+\alpha+\sigma}\left(1 - \frac{(\mu+\alpha)(\mu+\sigma)}{\kappa(\mu+\alpha+\sigma)}\right)\right] \\
&= -\frac{P(\mu+\sigma)}{(\mu+\alpha+\sigma)^2}\left[\left(1 - \frac{(\mu+\alpha)(\mu+\sigma)}{\kappa(\mu+\alpha+\sigma)}\right) + \frac{\kappa(\mu+\alpha+\sigma)(\mu+\sigma) - \kappa(\mu+\alpha)(\mu+\sigma)}{\kappa^2(\mu+\alpha+\sigma)}\right] \\
&= -\frac{P(\mu+\sigma)}{(\mu+\alpha+\sigma)^2}\left(1 - 2\frac{(\mu+\alpha)(\mu+\sigma)}{\kappa(\mu+\alpha+\sigma)} + \frac{\mu+\sigma}{\kappa}\right) \\
&= -\frac{P(\mu+\sigma)}{(\mu+\alpha+\sigma)^2}\left(\frac{\mu+\sigma}{\kappa}\left(1 - \frac{\mu+\alpha}{\mu+\alpha+\sigma}\right) + \left(1 - \frac{(\mu+\alpha)(\mu+\sigma)}{\kappa(\mu+\alpha+\sigma)}\right)\right) < 0,
\end{aligned}$$

since $\kappa > \frac{(\mu+\alpha)(\mu+\sigma)}{\mu+\alpha+\sigma}$ by the assumption that malaria is endemic.

Now, let $0 \leq \alpha_1 < \alpha_2$ and fix $\sigma \geq 0$. For notational convenience we omit the argument σ in the following calculation. From the calculation above we can further deduce that

$$S_m(\alpha_1) > S_m(\alpha_2) \Longrightarrow R_0^2(\alpha_1) = \frac{\beta^2\gamma S_m(\alpha_1)}{\rho(\mu+\alpha_1)H} > \frac{\beta^2\gamma S_m(\alpha_2)}{\rho(\mu+\alpha_2)H} = R_0^2(\alpha_2).$$

Finally, we are now in position to show the first claim:

$$\begin{aligned}
& h_e(\alpha_2) < h_e(\alpha_1) \\
\Leftrightarrow \quad & \frac{R_0^2(\alpha_2) - 1}{R_0^2(\alpha_2) + \beta/(\mu+\alpha_2)} < \frac{R_0^2(\alpha_1) - 1}{R_0^2(\alpha_1) + \beta/(\mu+\alpha_1)} \\
\Leftrightarrow \quad & 0 < (\mu+\alpha_1)(\mu+\alpha_2)(R_0^2(\alpha_1) - R_0^2(\alpha_2)) + \beta(R_0^2(\alpha_1)(\mu+\alpha_1) - R_0^2(\alpha_2)(\mu+\alpha_2)) + \beta(\alpha_2 - \alpha_1) \\
\Leftrightarrow \quad & 0 < (\mu+\alpha_1)(\mu+\alpha_2)(R_0^2(\alpha_1) - R_0^2(\alpha_2)) + \beta\frac{\beta^2\gamma}{\rho H}(S_m(\alpha_1) - S_m(\alpha_2)) + \beta(\alpha_2 - \alpha_1).
\end{aligned}$$

The former results show that the last line is true, hence the first claim follows. To see that the total number of mosquitoes $S_e + I_e + F_e$ also decreases with α, we calculate that

$$\begin{aligned}
\frac{\partial S_e + I_e + F_e}{\partial \alpha}(\alpha, \sigma) &= \frac{\partial}{\partial \alpha}\frac{\mu+\alpha+\sigma}{\mu+\sigma}S_m(\alpha, \sigma) \\
&= \frac{1}{\mu+\sigma}S_m(\alpha, \sigma) + \frac{\mu+\alpha+\sigma}{\mu+\sigma}\frac{\partial S_m}{\partial \alpha}(\alpha, \sigma) \\
&= \frac{P}{\mu+\alpha+\sigma}\left(1 - \frac{(\mu+\alpha)(\mu+\sigma)}{\kappa(\mu+\alpha+\sigma)}\right) \\
&\quad - \frac{P}{\mu+\alpha+\sigma}\left(\frac{\mu+\sigma}{\kappa}\left(1 - \frac{\mu+\alpha}{\mu+\alpha+\sigma}\right) + \left(1 - \frac{(\mu+\alpha)(\mu+\sigma)}{\kappa(\mu+\alpha+\sigma)}\right)\right) \\
&= -\frac{P\sigma(\mu+\sigma)}{\kappa(\mu+\alpha+\sigma)^2} \leq 0,
\end{aligned}$$

which only vanishes when $\sigma = 0$ and hence proves the second claim. □

Main Result 3 (Fungus-induced death rate on mosquitoes)**.** *For $\alpha > 0$ the total number of mosquitoes, $S_e + I_e + F_e$, decreases if the fungus-induced death rate σ increases.*

Proof. This is a straight forward calculation. Recall $S_e + I_e + F_e = S_m + F_m = S_m(1 + \frac{\alpha}{\mu+\sigma}) = P(1 - \frac{(\mu+\alpha)(\mu+\sigma)}{\kappa(\mu+\alpha+\sigma)})$ and calculate

$$\frac{\partial(S_e + I_e + F_e)}{\partial\sigma}(\alpha,\sigma) = -P\frac{\kappa(\mu+\alpha+\sigma)(\mu+\alpha) - \kappa(\mu+\alpha)(\mu+\sigma)}{\kappa^2(\mu+\alpha+\sigma)^2} = -\frac{P\alpha(\mu+\alpha)}{\kappa(\mu+\alpha+\sigma)^2},$$

which is negative for all $\alpha > 0$. □

Main Result 4 (Fungus-induced death rate on malaria prevalence in humans)**.** *If $\alpha = 0$ (i.e. no fungus spraying) the human malaria incidence rate $h_e = h_e(0,\sigma)$ is constant in σ. If $\alpha > 0$ (i.e. the fungus is applied) the malaria prevalence in the human population $h_e(\alpha,\sigma)$, as well as the total number of infected mosquitoes $I_e(\alpha,\sigma)$, both have their maximum at*

$$\sigma^* = \frac{(\kappa - 2\mu)(\mu+\alpha)}{(2\mu-\kappa)+2\alpha}.$$

Proof. Let $\alpha \geq 0$ be fixed. To find the local maximum of $h_e(\alpha,\sigma)$ we first calculate the derivative with respect to σ. This yields

$$\frac{\partial h_e}{\partial\sigma}(\alpha,\sigma) = \frac{1+\beta/(\mu+\alpha)}{(R_0^2(\alpha,\sigma)+\beta/(\mu+\alpha))^2}\frac{\partial R_0^2}{\partial\sigma}(\alpha,\sigma)$$

so that we see that $h_e(\alpha,\sigma)$ has a critical point whenever $R_0^2(\alpha,\sigma)$ has a critical point. However, $R_0^2(\alpha,\sigma)$ in turn a critical point whenever $S_m(\alpha,\sigma)$ has a critical point:

$$\frac{\partial R_0^2}{\partial\sigma}(\alpha,\sigma) = \frac{\beta^2\gamma}{\rho(\mu+\alpha)H}\frac{\partial S_m}{\partial\sigma}(\alpha,\sigma).$$

Thus we first study the critical points of $S_m(\alpha,\sigma)$.

$$\begin{aligned}\frac{\partial S_m}{\partial\sigma}(\alpha,\sigma) &= P\left[\frac{\alpha}{(\mu+\alpha+\sigma)^2}\left(1-\frac{(\mu+\alpha)(\mu+\sigma)}{\kappa(\mu+\alpha+\sigma)}\right) - \frac{\mu+\sigma}{\mu+\alpha+\sigma}\frac{(\mu+\alpha+\sigma)(\mu+\alpha)-(\mu+\sigma)(\mu+\alpha)}{\kappa(\mu+\alpha+\sigma)^2}\right]\\ &= \frac{P\alpha}{(\mu+\alpha+\sigma)^2}\left(1-2\frac{(\mu+\alpha)(\mu+\sigma)}{\kappa(\mu+\alpha+\sigma)}\right).\end{aligned}$$

Now we see that if $\alpha = 0$, then $S_m(0,\sigma)$, and hence $h_e(0,\sigma)$ is constant for all values of σ. So from now on let $\alpha > 0$. Then the value σ^* that makes $\frac{\partial}{\partial\sigma}S_m(\alpha,\sigma^*) = 0$ solves

$$\begin{aligned} & 1 = 2\frac{(\mu+\alpha)(\mu+\sigma^*)}{\kappa(\mu+\alpha+\sigma^*)}\\ \Leftrightarrow \quad & \sigma^*(\kappa - 2(\mu+\alpha)) = (\mu+\alpha)(2\mu-\kappa)\end{aligned}$$

If $\kappa = 2(\mu+\alpha)$, then the equation above yields $0 = (\mu+\alpha)\alpha$, which is only true for $\alpha = 0$, since $\mu > 0$. As we have asserted before, h_2 is constant in this case. Therefore, if $\kappa = 2(\mu+\alpha)$ no maximum exists. If $\kappa \neq 2(\mu+\alpha)$ we obtain

$$\sigma^* = \frac{(\kappa-2\mu)(\mu+\alpha)}{(2\mu-\kappa)+2\alpha},$$

i.e. $\frac{\partial S_m}{\partial\sigma}(\alpha,\sigma^*) = 0$ and thus $\frac{\partial R_0^2}{\partial\sigma}(\alpha,\sigma^*) = 0 = \frac{\partial h_e}{\partial\sigma}(\alpha,\sigma^*)$. We use the second derivative test to quickly check that the value at (α,σ^*) is indeed a maximum:

$$\begin{aligned}\frac{\partial^2 S_m}{\partial\sigma^2}(\alpha,\sigma^*) &= \frac{2P\alpha}{(\mu+\alpha+\sigma^*)^3}\left(\overbrace{-1+2\frac{(\mu+\alpha)(\mu+\sigma^*)}{\kappa(\mu+\alpha+\sigma^*)}}^{=0} - \frac{(\mu+\alpha+\sigma^*)(\mu+\alpha)-(\mu+\sigma^*)(\mu+\alpha)}{\kappa(\mu+\alpha+\sigma^*)}\right)\\ &= -2\frac{P\alpha(\mu+\alpha)}{\kappa(\mu+\alpha+\sigma^*)^3}\left(1-\frac{\mu+\sigma^*}{\mu+\alpha+\sigma^*}\right) < 0.\end{aligned}$$

Since $\frac{\partial S_m}{\partial \sigma}(\alpha, \sigma)$ and and $\frac{\partial h_e}{\partial \sigma}(\alpha, \sigma)$ have the same sign everywhere this implies that h_e also has its maximum at (α, σ^*). Finally, I_e is an increasing function of h_e and hence is also maximized at (α, σ^*). $\square$
