# Supplementary material for: Assessing the optimal virulence of malaria‐targeting mosquito pathogens: a mathematical study of engineered Metarhizium anisopliae
Source: Malar J. 2014 Jan 8;13:11. doi: 10.1186/1475-2875-13-11 (PMC3893513; doi:10.1186/1475-2875-13-11)
Supplement: Additional file 2 — Includes details of parameter estimation used in generating the figures. [file 1475-2875-13-11-S2.pdf]

Additional File 2 for: Assessing the optimal virulence of malaria-targeting mosquito pathogens: a mathematical study of engineered *Metarhizium anisopliae*

Bernhard Konrad[1], Michael Lindstrom[1], Anja Gumpinger[2], Jielin Zhu[1], Daniel Coombs[1,*]

1 Department of Mathematics and Institute of Applied Mathematics, University of British Columbia, Vancouver, BC, Canada

2 TU München, Fakultät für Mathematik, Boltzmannstraße 3, 85748 Garching (b. München), Germany

[*]E-mail: coombs@math.ubc.ca

Parameters

We choose some reasonable parameter values to illustrate our analytical results. Consider a small village with constant population size of $H = 3000$ humans. It is fair to assume that female mosquitoes typically outnumber humans by a factor of three to ten (see [Chi08, Smi08]), hence we choose the carrying capacity of mosquitoes to be $\tilde{P} = 10000$. About half of bites from infectious mosquitoes lead to an infection in a human [Chi06, Chi08], i.e. $\gamma = 0.5$.

The biting rate β is difficult to measure as it depends on many factors, in particular (seasonal) human behavior. In our simple model we assume a constant mass-action rate. The value used in [Smi08], $\beta\gamma\tilde{P}/H = 0.5$ would translates to $\beta = 0.3$ day^{-1}, which is about the maximal number of times one mosquito would want to bite humans per unit time, if humans were freely available, see [Chi06]. Since it is reasonable to assume that humans are somewhat not always freely available in a village we choose $\beta = 0.15$ days^{-1}. This choice does not significantly affect the qualitative results of the study. Infected humans leave the infectious class by natural or malaria-induced death or when they develop (temporal) immunity. The latter is the dominating contributor, and usually happens after a few months to a year, hence we choose an average of 200 days, i.e. $\rho = 0.005$ days^{-1} (see [Koe03, Chi06]). The mean life-time of an outgrown, biting, female mosquito is around ten days [Koe03, Smi08] and hence the mosquito death rate is chosen to be $\mu = 0.1$ days^{-1}.

A typical mosquito population is not constant throughout a year but is heavily influenced by external factors such as rainfall and humidity. A small mosquito population at end of a dry season typically experiences a fast growth at the beginning of the rain season. Here is field data from [Ade10], where the number of mosquitoes caught in a trap close to a village in Nigeria was measured between August 2005 and July 2006:

	Aug	Sep	Oct	Nov	Dec	Jan	Feb	Mar	Apr	May	Jun	Jul
Anopheles gambiae	23	24	25	21	9	5	12	26	47	50	76	46

The growth phase from January until June can clearly be seen. We assume that, in this time period, the environmental conditions are so optimal for the mosquito population, that it experiences exponential growth. So we let $M = S + I$ be the total number of mosquitoes, set $\alpha = 0$, hence $F = 0$, since in the data no fungus was applied, and fit the data points above to the simplified model

$$\frac{dM}{dt} = (\kappa - \mu)M, \qquad M(0) = M_0.$$

Then we perform a least-square fit optimization for $(\kappa - \mu)$ and M_0 to obtain $(\kappa - \mu) \approx 0.38$ days^{-1}, or simply $\kappa = 0.48$ days^{-1}.

In the life-stage-structured model we need to estimate the larval production rate κ_c and intra-specific larval competition parameter κ_L. For simplicity, we set $\kappa_c = \kappa$ as estimated above, and then choose κ_L so that the number of mosquitoes is equal to the carrying capacity in the absence of the fungus.

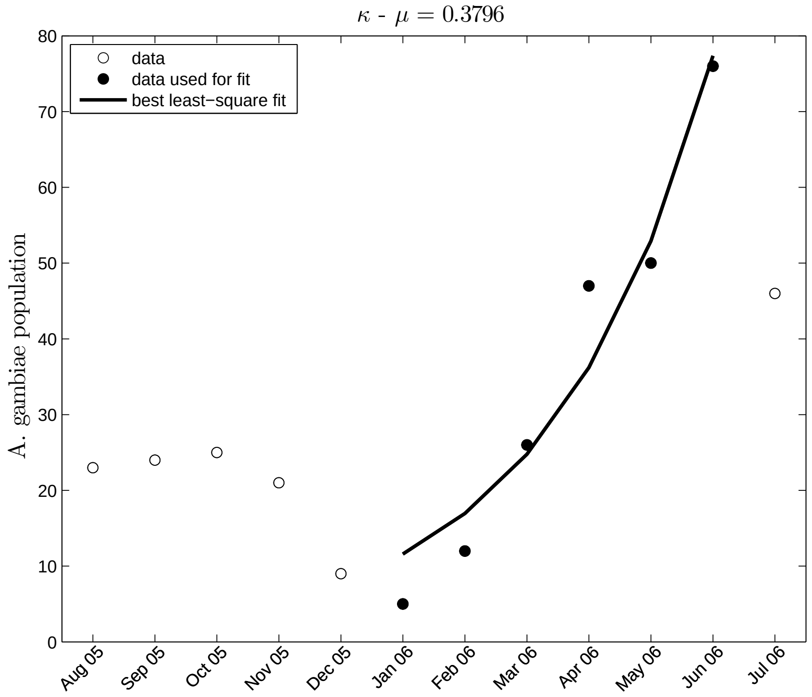

Figure 1: **Data fitting for κ.** We fit an exponential function for six of the data points reported in [Ade10] to find an approximation for $\kappa - \mu$.

Symbol	Value	Meaning	Unit	Source
H	3000	Total human population	humans	-
$\tilde{P}$	10000	Mosquito carrying capacity	mosquitoes	[Chi06, Smi08]
γ	0.5	proportion of infectious bites from an infected mosquito	-	[Chi06, Chi08]
β	0.15	per capita mosquito biting rate	days^{-1}	[Chi06]
ρ	0.005	recovery rate of infected humans	days^{-1}	[Koe03, Chi06]
μ	0.1	Natural mosquito death rate	days^{-1}	[Koe03, Smi08]
κ	0.48	Innate mosquito growth rate	days^{-1}	[Ade10]
α	varied	Exposure rate of the fungus	days^{-1}	-
σ	varied	Fungus-induced death rate	days^{-1}	-
ξ	varied	Vertical transmissibility of fungus	days^{-1}	-
κ_c	0.48	Mosquito larvae birth rate	days^{-1}	see text
κ_L	3.8×10^{-5}	Larval competition parameter	days^{-1} larvae^{-1}	see text

Table 1: Table of parameter estimates

References

[Ade10] **M.A. Adelekea, C.F. Mafianaa, A.B. Idowua, S.O. Sam-Woboa, O.A. Idowua.** *Population dynamics of indoor sampled mosquitoes and their implication in disease transmission in Abeokuta, south-western Nigeria.* J Vector Borne Dis 47, March 2010, pp. 33-38.

[Chi06] **N. Chitnis, J.M. Cushing, J.M. Hyman.** *Bifurcation analysis of a mathematical model for malaria transmission.* SIAM J. Appl. Math. Vol 67, No. 1, pp. 24-45, doi:10.1137/050638941.

[Chi08] **N. Chitnis, J.M. Hyman, J.M. Cushing.** *Determining Important Parameters in the Spread of Malaria Through the Sensitivity Analysis of a Mathematical Model.* Bulletin of Mathematical Biology (2008), 70:1272-1296, doi:10.1007/s11538-008-9299-0.

[Koe03] **J.C. Koella, R. Antia.** *Epidemiological models for the spread of anti-malarial resistance.* Malaria Journal 2003, 2:3, doi:10.1186/1475-2875-2-3, http://www.malariajournal.com/content/2/1/3.

[Smi08] **R.J. Smith? and S.D. Hove-Musekwa.** *Determining Effective Spraying Periods to Control Malaria via Indoor Residual Spraying in Sub-Saharan Africa.* Journal of Applied Mathematics and Decision Sciences Volume 2008, Article ID 745463, doi:10.1155/2008/745463.
